# On Secure Simple Pairing in Bluetooth Standard v5.0-Part II: Privacy Analysis and Enhancement for Low Energy

**DOI:** 10.3390/s19153259

**Published:** 2019-07-24

**Authors:** Da-Zhi Sun, Li Sun, Ying Yang

**Affiliations:** 1 Tianjin Key Laboratory of Advanced Networking (TANK), Division of Intelligence and Computing, Tianjin University, No. 135, Yaguan Road, Tianjin Haihe Education Park, Tianjin 300350, China; 2 Standardization Department, China Aero-Polytechnology Establishment, Aviation Industry of China, No. 7, Jingshun Road, Chaoyang District, Beijing 100028, China

**Keywords:** Bluetooth standard, low energy, secure simple pairing, cryptographic protocol, untraceability, privacy model

## Abstract

Bluetooth low energy devices are very popular in wireless personal area networks. According to the Bluetooth standard specifications, the low energy secure simple pairing (LESSP) protocol is the process by which the pairing devices negotiate the authenticated secret key. To violate the user privacy, the adversary can perhaps link the runs of the LESSP protocol to the targeted device, which usually relates to the specially appointed user. Hence, we investigate deep into the privacy of the LESSP protocol. Our main contributions are threefold: (1) We demonstrate that the LESSP protocol suffers from privacy vulnerability. That is, an adversary without any secret key is able to identify the targeted device by the LESSP protocol. (2) An improvement is therefore proposed to repair the privacy vulnerability in the LESSP protocol. (3) We develop a formal privacy model to evaluate the privacy vulnerabilities in the LESSP protocol and its improved versions. We further prove that our improvement on the LESSP protocol is private under the privacy model. In addition, the performance evaluation shows that our improvement is as efficient as the LESSP protocol. Our research results are beneficial to the privacy enhancement of Bluetooth systems in wireless personal area networks.

## 1. Introduction

Due to the development of ubiquitous computing, more and more people carry networking devices, e.g., laptops, smartphones, tablets, and smart watches. These devices are always interconnected via wireless short-range radio frequency communications such as Bluetooth and Wi-Fi. In ubiquitous computing environments, low energy (LE) is an important but challenging requirement for wireless and mobile devices. As the crucial innovation of Bluetooth, the LE form aims to provide low-power and low-cost wireless communications. The LE form was first introduced in Bluetooth standard v4.0 [1] and improved later in Bluetooth standard v4.2 [2]. Bluetooth standard v5.0 [3] further enhances Bluetooth standard 4.2’s LE form with up to 4× the range, 2× the speed, and 8× the broadcasting message capacity [4]. Therefore, the LE form in Bluetooth standard v5.0 is treated as an ideal solution of the Internet of Things (IoT) and has been widely deployed in personal or commercial applications, compared with its basic rate/enhanced data rate (BR/EDR) form. The very recent Bluetooth standard v5.1 [5] still maintains the same LE design as Bluetooth standard v5.0.

**Figure 1:**
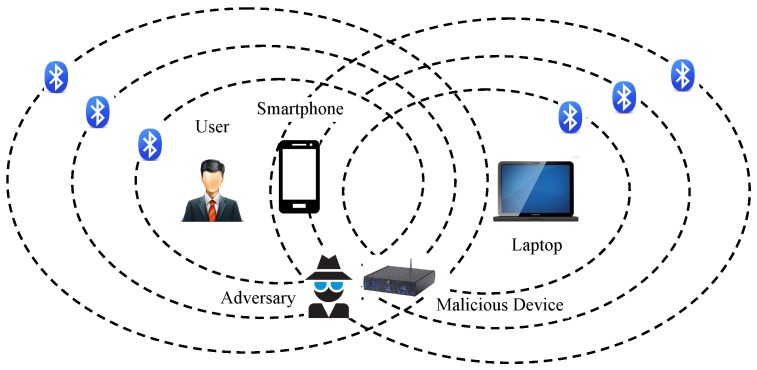
Privacy threat scenario in a typical Bluetooth application.

Bluetooth LE devices have flourished in various person-related fields such as wireless personal area networks. However, these devices may incur severe privacy threats to the users, that is, the adversary can track users’ identities through the devices. Consider the scenario where the user connects his smartphone to his nearby laptop via the Bluetooth LE channel. As shown in Figure 1, the adversary can identify the user’s identity with his malicious device, because the Bluetooth LE channel is susceptible to radio signal interception. More importantly, the user’s exposed identity can be further used as a clue to disclose more sensitive personal information such as medical conditions, financial details, religion preferences, or privileged communications with lawyers. Therefore, a privacy mechanism should be introduced to prevent illegal traceability caused by exploiting the wireless communication processes of the devices.

### 1.1. Bluetooth LE Security and Privacy

The pairing is the core design to realize the security services in the LE form. In detail, the pairing establishes the secret keys between two Bluetooth devices, and then encrypts a link of the Bluetooth devices and authenticates the Bluetooth devices by using these keys. The pairing method in Bluetooth standards v4.0 and v4.1 [1,6] is referred to as LE legacy pairing. The subsequent Bluetooth standards [2,3,5] adopt LE secure connections pairing as an enhanced version. LE secure connections pairing can be treated as LE secure simple pairing (LESSP). The pairing procedure consists of three phases as follows:

- Phase 1: Pairing feature exchange.
- Phase 2: LESSP protocol or LE legacy pairing protocol.
- Phase 3: Transport specific key distribution (optional).

The pairing feature exchanged in phase 1 determines which of two pairing methods shall be used in phase 2. By default, a LESSP protocol is run in phase 2. A LE legacy pairing protocol is also executable if necessary. Meanwhile, when the LESSP protocol is used, phase 1 needs to choose one of the association models according to the input, output, and display capability. The available association models are the numeric comparison (NC) model, the out of band (OOB) model, the passkey entry (PE) model, and the just works (JW) model. Optionally, phase 3 may be performed to distribute transport specific keys, for example, the identity resolving key (IRK) value.

We can outline the security features of the pairing methods. Since LE legacy pairing has no NC model and does not use the elliptic curve Diffie-Hellman (ECDH) algorithm, it cannot prevent passive eavesdropping except when the OOB model is used. Comparatively, LESSP employs not only the ECDH algorithm to protect from the passive eavesdropping but also the NC, OOB, and PE models to prevent man-in-the-middle (MITM) attacks. Hence, the National Institute of Standards and Technology (NIST) [7] recommends LESSP. However, LESSP should further provide the privacy protection due to the fast development of the personal Bluetooth applications. It is meaningful to ensure that the adversary is unable to identify, track, and associate the targeted devices by exploiting the runs of the pairing protocol. Hence, we will investigate the privacy of LESSP according to Bluetooth standards v5.0 and v5.1 [3,5].

### 1.2. Related Work

The privacy of the Bluetooth LE form has been widely studied. Gomez et al. [8] described that the LE form offers privacy feature and mitigates the risk that the devices will be tracked by an adversary. Gibbs [9] presented a technical guide to the basis of Bluetooth LE privacy. Wang [10] realized a privacy enhancement protocol over the advertising channels of the LE form. Raza et al. [11] introduced the enhanced privacy feature of the LE form in Bluetooth standard v4.2 and implemented it in the IoT devices. Fawaz et al. [12] investigated the privacy threats from the Bluetooth LE devices and proposed a guardian system to protect the privacy of the users equipped with these devices. AN99209 [13] provided a privacy overview of the link layer of the LE form and compared the privacy features in Bluetooth standards v4.1 and v4.2. Hassan et al. [14] investigated some security and privacy threats in the LE form and the BR/EDR form, such as the pin theft attack, the eavesdropping attack, and the victim device cloning attack. The privacy in the applications of the Bluetooth LE form has also been focused on. Mare et al. [15] proposed a hide-n-sense privacy system, which uses the LE form as a crucial component of the wireless access. Cyr et al. [16] showed that the Fitbit Flex ecosystem provides a reasonable level of privacy for user data, when the adversary observes the LE traffic between the Fitbit devices. Das et al. [17] studied the possible privacy leakage from Bluetooth LE communications between the fitness tracker and the smartphone, i.e., tracking the user location via unchanged Bluetooth LE address and inferring user’s current activity through Bluetooth LE traffic analysis. Korolova et al. [18] investigated the possibilities of cross-app tracking using nearby Bluetooth LE devices on Android and iOS. Sivakumaran et al. [19] showed how unauthorized co-located Android applications can access pairing-protected LE data and presented the results of a large-scale static analysis over 18,900 LE Android applications. Cha et al. [20] deployed a privacy framework where the smartphones access the nearby IoT devices via the LE channel. Despite the increasing interest in the investigation of the LESSP security [21,22,23,24], previous work did not focus on the LESSP privacy. We examine the LESSP privacy under all possible means of privacy infringement, because the adversary must be expected to exploit any available privacy defects existing in the Bluetooth service.

Moreover, we note that the privacy of the Radio Frequency Identification (RFID) protocols has been well addressed under the formal method. For example, Ha et al. [25] and Juels and Weis [26] proposed formal privacy definitions for the RFID protocols and used them to evaluate the privacy of the RFID protocols. In light of the applicability of RFID-based privacy solutions, Kostakos [27] explored Bluetooth privacy implications. In fact, as the state-of-the-art pairing protocols [28,29,30], the lack of the formal treatment inspires us to formally analyze the privacy of the LESSP protocol.

### 1.3. Our Contribution

The LESSP protocol is responsible for the negotiation of the authenticated secret keys between the pairing devices, which usually relate to the specially appointed users. The privacy protection requires that the targeted device’s runs of the LESSP protocol be not linked by the adversary. We address this privacy problem on the LESSP protocol. Our main contributions are threefold: (1) We demonstrate that the LESSP protocol suffers from privacy vulnerability, that is, an adversary without any secret key can identify the targeted device, when the targeted device runs the LESSP protocol. (2) An improvement is therefore proposed to repair the existing privacy vulnerability in the LESSP protocol. (3) A formal privacy model is developed to evaluate the privacy of the LESSP protocol and its improved versions. We further prove that our improvement on the LESSP protocol is private under our privacy model. In addition, the performance evaluation shows that our improvement is as efficient as the LESSP protocol.

To the best of our knowledge, previous work [22,28,29,30] seldom involves the formal model, which can evaluate the security and privacy of the SSP protocol and the LESSP protocol. In Part I [31], we therefore propose a formal security model for the SSP protocol. In this part, a formal privacy model is also presented to define the privacy feature for the LESSP protocol. It needs to be pointed out that the substantial contribution in this part is not only a privacy enhancement on the LESSP protocol but also a privacy model which can be reused to evaluate the privacy of the follow-up LESSP protocols in the future Bluetooth standards. Although the LESSP protocol and the SSP protocol have a slight difference, these two formal models are suitable to evaluate both of them.

### 1.4. Notation

In Table 1, we list the notations used throughout the rest of the paper. Other notations will be defined where they are first used.

## 2. LESSP Protocol

### 2.1. Overview

**Figure 2:**
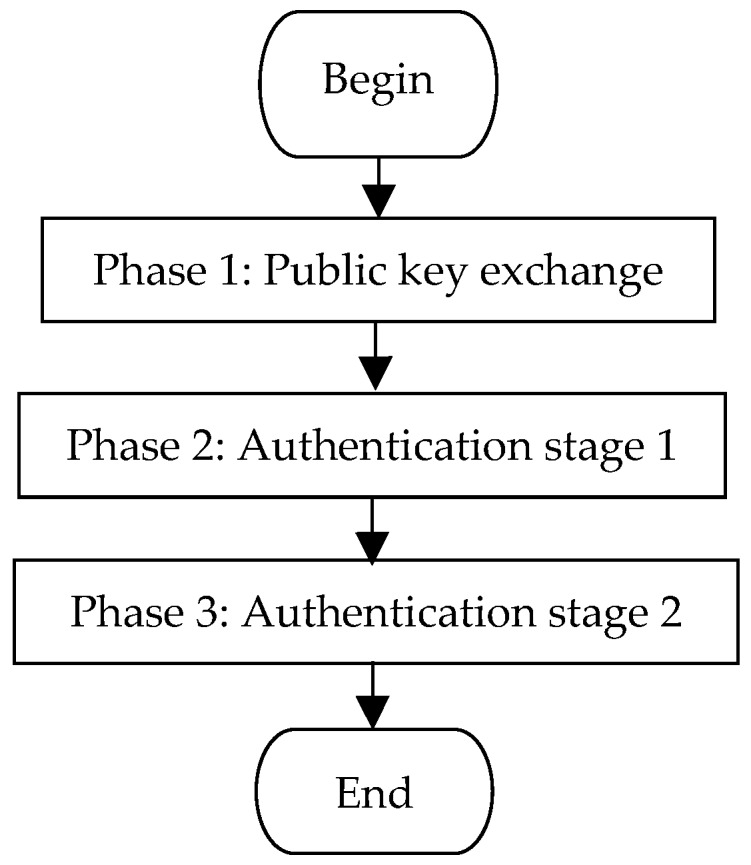
Flowchart of LESSP protocol.

Π* aims to establish the authenticated *LTK* between the pairing Bluetooth devices. *LTK* is subsequently used in the authentication process and the sensitive data transmission process. As shown in Figure 2, Π* consists of three consecutive phases:

Phase 1: Public key exchange. In this phase, both devices exchange their public keys.
Phase 2: Authentication stage 1. This phase is used to prevent MITM attacks by running one of the three association models, i.e., the NC, OOB, or PE model. This phase also outputs the random nonces for the next phase.
Phase 3: Authentication stage 2. Both devices in this phase confirm that they have generated *MacKey* and *LTK* and successfully completed the run of Π*.

### 2.2. Detailed Description

**Figure 3:**
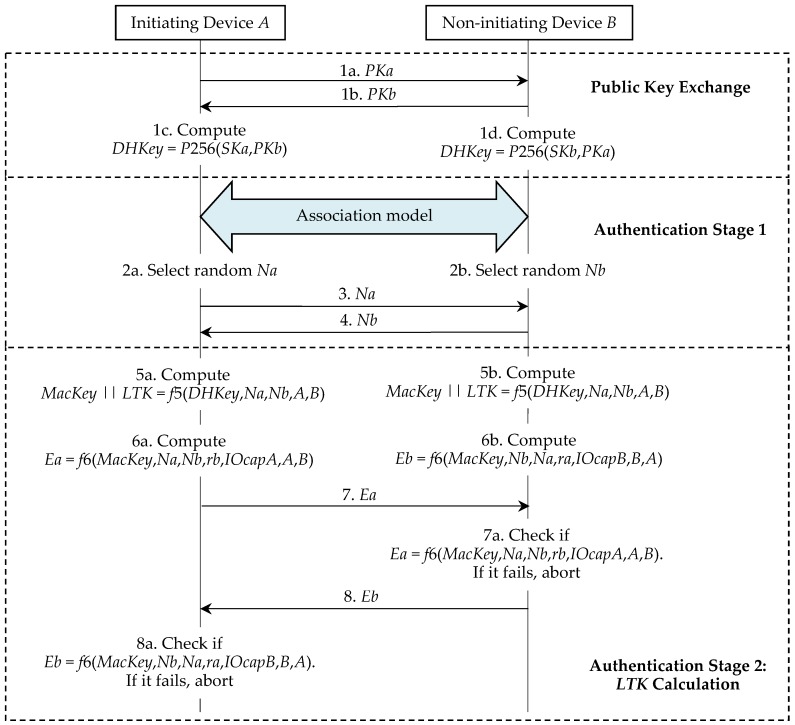
LESSP protocol Π*.

Initially, each device generates its own ECDH private-public key pair. That is, the device *A* and the device *B* generate (*SKa*, *PKa*) and (*SKb*, *PKb*) respectively. Bluetooth standard v5.0 [3], p. 2318 states that: “This key pair needs to be generated only once per device and may be computed in advance of pairing. A device may, at any time, choose to discard its public-private key pair and generate a new one, although there is not a requirement to do so.” Π* is illustrated in Figure 3 and we further describe it as follows.

#### 2.2.1. Phase 1. Public Key Exchange

The initiating device *A* starts a run of Π* and sends its *PKa* to the responding device *B* (step 1a). The responding device *B* replies with its *PKb* (step 1b). The device *A* and the device *B* compute their shared *DHKey* (step 1c and step 1d).

#### 2.2.2. Phase 2. Authentication Stage 1

This phase implements one of the four association models, i.e., the NC, OOB, PE, or JW model. To prevent MITM attacks, the NC, OOB, and PE models require that the user take part in this authentication process. The JW model is the same as the NC model, except that the JW model does not require the user to confirm the commitment checks in both devices. Hence, the JW model cannot prevent MITM attacks. We omit the review of the details of the association models, because the privacy problem in these models will not be discussed. No matter what the association model is used, the device *A* and the device *B* respectively generate the random nonce *Na* and the random nonce *Nb*, and then exchange *Na* and *Nb* for the next phase. Hence, we separate these processes (step 2a, step 2b, step 3, and step 4) from the association models. In addition, Π* requires that both *ra* and *rb* be all set to 0 in the NC and JW models, that both devices generate and exchange the random *ra* and *rb* in the OOB model, and that the user in the PE model enter the same *ra* and *rb* into the devices.

#### 2.2.3. Phase 3. Authentication Stage 2

Each device computes *MacKey* and *LTK* using the shared *DHKey* and the previously exchanged values (step 5a and step 5b). Here, || denotes the bit string concatenation and *A* and *B* are secretly exchanged before. Then the device *A* and the device *B* respectively compute *Ea* and *Eb* by using the newly shared *MacKey* (step 6a and step 6b). Here, *IOCapA* and *IOCapB* are negotiated by the devices during phase 1 of the pairing procedure. The device *A* then transmits its *Ea* to the device *B* (step 7). If the device *B*’s check fails, it indicates that the device *A* has not confirmed the pairing run’s authenticity and the pairing run must be aborted by the device *B* (step 7a). The device *B* then transmits its *Eb* to the device *A* (step 8). A failure check also means that the device *B* has not confirmed the pairing run’s authenticity and the pairing run should be aborted by the device *A* (step 8a).

## 3. Privacy Vulnerability on LESSP Protocol

The Bluetooth LE specifications provide some privacy features. For example, the Bluetooth LE solution supports the random resolvable private address (RPA) concept, when the devices need the privacy protection services. The random RPA is generated using IRK and updated after a given interval. And the trusted peer devices resolve the random RPA to an identity address such as the public address or the static address of the device, instead of transmitting the identity address directly. All devices maintain a list of the peer device’s identity addresses, the local IRK used to calculate its own random RPA, and the peer device’s IRK used to resolve the peer device’s random RPA. Due to the random RPA, the adversary is not easy to track the device by its identity address. However, we find that the device can be tracked by exploiting the runs of Π*.

### 3.1. Privacy Attacks

Each pairing device must transmit its own ECDH public key over the LE channel during every run of Π*. The Bluetooth LE specification allows that the ECDH private-public key pair is generated only once per device and is reused in the multiple runs of Π*. Hence, in order to track the device, the adversary is able to collect these public keys transmitted in each run of Π* and further identify the corresponding runs with a certain device. In detail, the adversary can perform the following steps to track a device *X* via the runs of Π*:

Step 1. The adversary marks the targeted device *X*. In the device *X*’s run of Π*, the adversary eavesdrops on its *PKx* during the public key exchange phase and stores it.
Step 2. In the follow-up runs of Π*, the adversary eavesdrops on each public key during the public key exchange phase. And then, the adversary checks whether the current public key is equal to his local *PKx* stored in step 1. If so, the adversary knows the device *X* takes part in the corresponding run and records the counterpart device.

**Figure 4:**
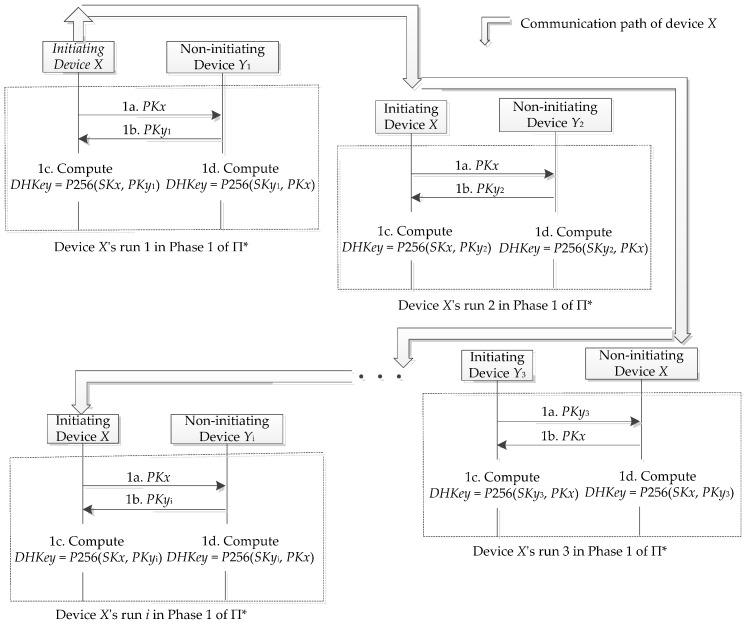
An example of the tracked device *X*’s communication profile.

Figure 4 shows how to create the device *X*’s communication profile by using the above privacy attack. This profile potentially discloses the Bluetooth activities of the device *X* until the change of its *PKx*. A sophisticated adversary can build up a communication constellation for multiple devices by extending the above privacy attack as follows.

Step 1. The adversary marks the multiple targeted devices, and then collects and stores the corresponding public keys in step 1 of the above privacy attack.
Step 2. In step 2 of the above privacy attack, the adversary eavesdrops on the public keys in the subsequent runs of Π* and instead compares them with each of the collected public keys in step 1.

**Figure 5:**
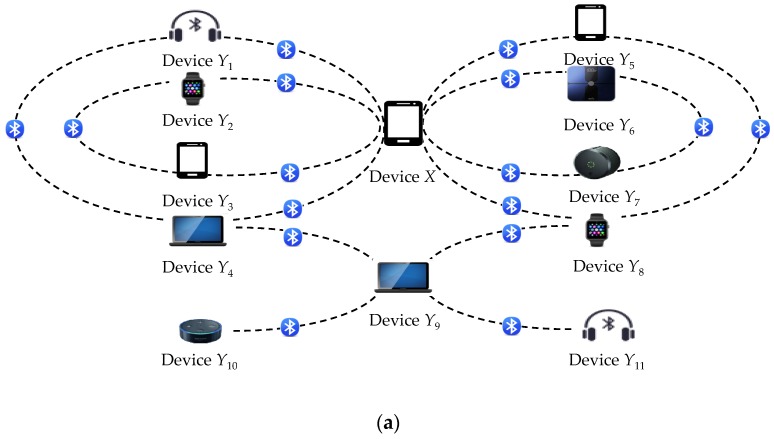

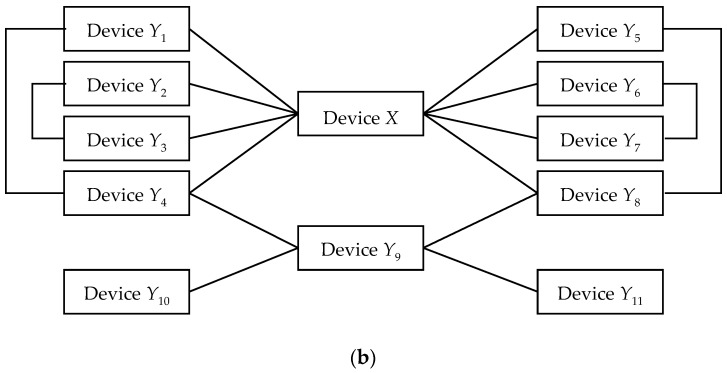
An example of the extended privacy attack: (**a**) The communication activities of multiple tracked devices; (**b**) The tracked devices’ communication constellation derived from Figure 5a.

Figure 5 gives an example of the extended privacy attack. This extension helps the adversary to infer the user’s behavior characteristics and the multiple users relationship via a great deal of the tracked devices.

### 3.2. Technical Remarks and Experimental Confirmation

The proposed privacy attacks are practical due to the following technical factors:

1. To defend itself against the proposed attacks, the device may update its public key in each run of Π*. We argue that the device tends to reuse the public key during the multiple runs of Π*. The device needs to expend more system resources, especially more device energy, if the ECDH private-public key pair is frequently updated. However, the device energy cost is just the critical factor in the LE form.
2. The proposed attacks merely exploit the vulnerability in the public key exchange phase of Π*. We know that the public key exchange phase is the same no matter what the NC, OOB, PE, or JW model is selected. Hence, the proposed attacks are regarded as the broad-spectrum tracking ways.
3. The proposed attacks can be performed in the off-the-shelf Bluetooth tools without the need for any hardware retrofit, because they only eavesdrop on the transmitted messages over the LE channel.

**Figure 6:**
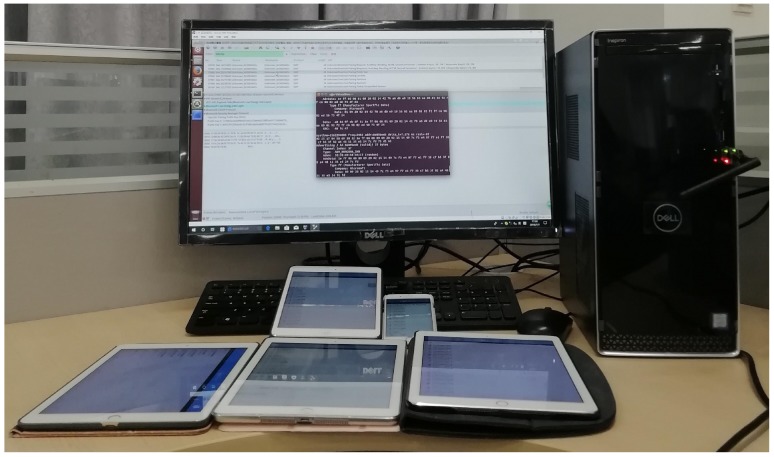
Privacy experiment platform for LE Bluetooth devices.

**Figure 7:**
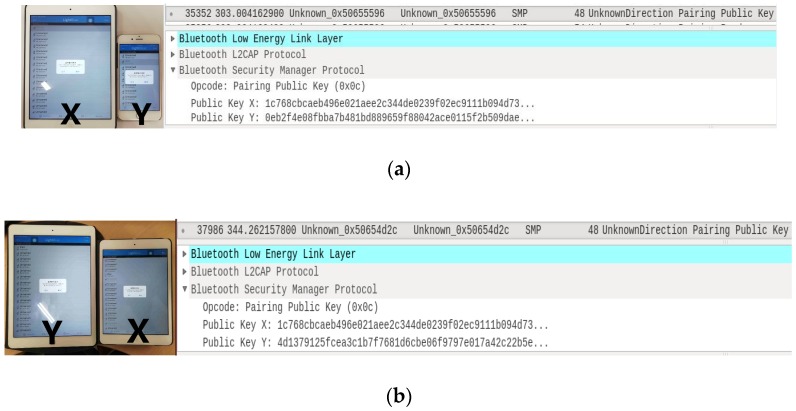
(**a**) Test 1 and its corresponding pairing public key packet (public key *X*: 1c768cbcaeb496e 021aee2c344de0239f02ec9111b094d7302d017175e50b4a5, public key *Y*: 0eb2f4e08fbba7b481bd8 89659f 88042ace0115f2b509dae3c8c15510a37d5ba); (**b**) Test 2 and its corresponding pairing public key packet (public key *X*: 1c768cbcaeb496e021aee2c344de0239f02ec9111b094d7302d017175e50b4a5, public key *Y*: 4d1379125fcea3c1b7f7681d6cbe06f9797e017a42c22b5e4a30ce5841fb73d6).

Moreover, as shown in Figure 6, we build an experiment platform to verify the proposed attacks. We use a commercially available sniffer Ubertooth One USB dongle under the Ubuntu 14.04.1 in the Oracle VM VirtualBox and test the LESSP protocol implemented by the LightBlue apps of iOS devices. Figure 7 shows the pairing public key packets sniffed by Ubertooth One USB dongle during two runs of the LESSP protocol. Here, we can track the device *X*.

## 4. Improvement on LESSP Protocol

### 4.1. Design Frame

**Figure 8:**
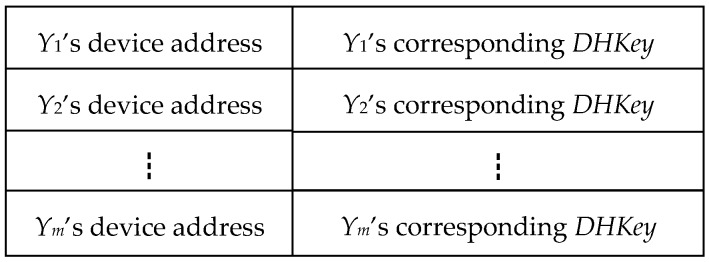
The device *X*’s pairing information table.

Informally, the adversary fails to track the device *X* by implementing the proposed attacks, if the device *X* randomly generates a new private-public key pair (*SKx*, *PKx*) for every run of Π*. However, this update operation is inefficient. To securely reuse the key pair, our idea is to create a pairing information table, that is, each device *X* secretly maintains a table for its previous pairing runs. As shown in Figure 8, we require that each device *X*’s table store its previous pairing information with the devices *Y*_1_, *Y*_2_, …, and *Y_m_*. Each record in the table needs to contain the device address and the corresponding *DHKey*. Once any a record compromises or expires, the device *X* can remove it from its table. Certainly, the device *X* should first choose the information of the frequently paired devices into the table.

**Figure 9:**
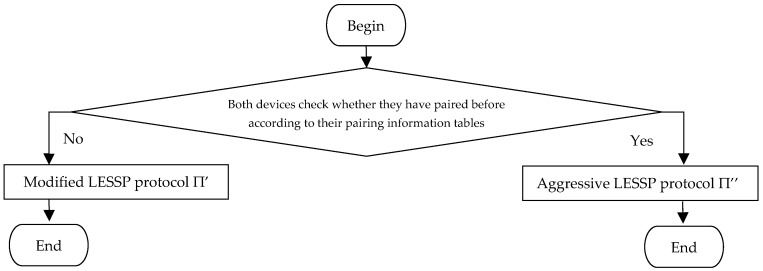
Flowchart of our improvement (Π′, Π″).

When any two devices *X* and *Y* begin to pair, they first search their pairing information tables indexed the device address. If both devices find the other one in their pairing information tables, then they implement an aggressive LESSP protocol Π″; else they implement a modified LESSP protocol Π′, where Π′ and Π″ are respectively proposed in the following two subsections. Figure 9 describes the process flow of (Π′, Π″). Here, similar to Π*, both Π′ and Π″ should implement the association model to verify the public keys. However, for simplicity, we omit it, because the privacy of the association model is not considered.

### 4.2. Modified LESSP Protocol

**Figure 10:**
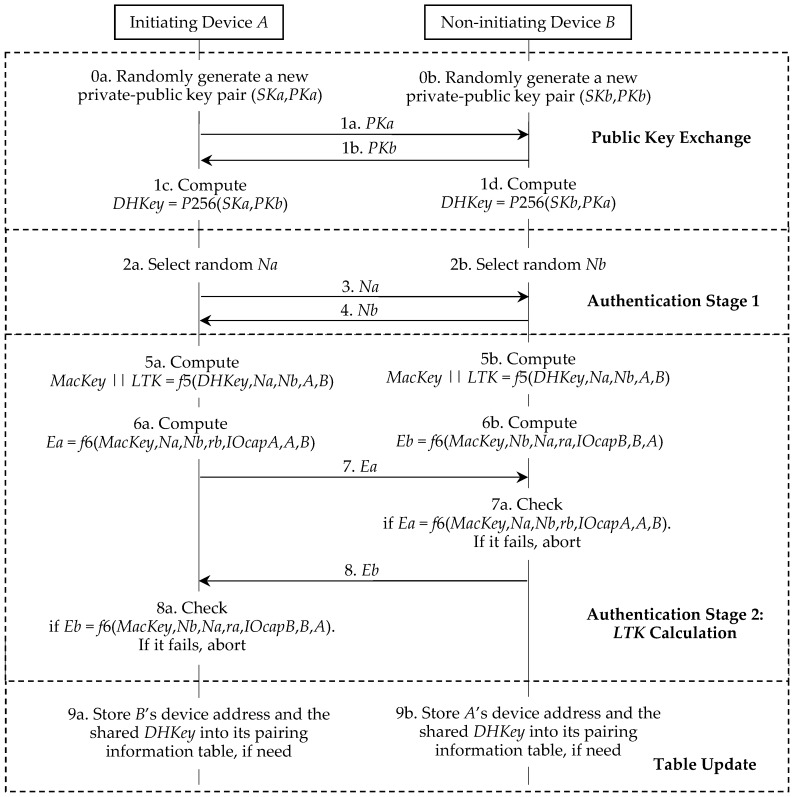
Modified LESSP protocol Π′.

As shown in Figure 10, Π′ is almost the same as Π* in Figure 3. The different points of two protocols are as follows:

(1) Π′ mandatorily requires that the device *A* and the device *B* randomly generate the new private-public key pairs.
(2) In the table update phase of Π′, the device *A* and the device *B* may store new data into their pairing information tables.

### 4.3. Aggressive LESSP Protocol

**Figure 11:**
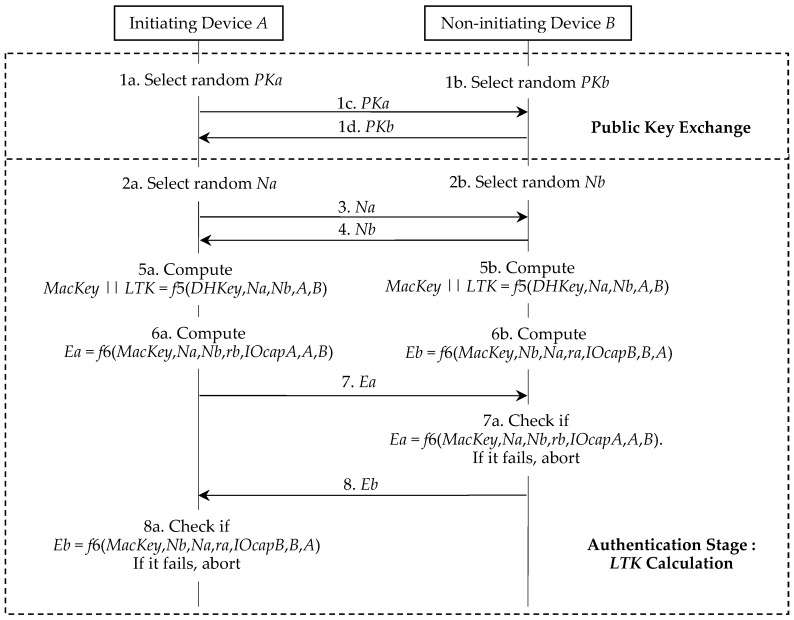
Aggressive LESSP protocol Π″.

As shown in Figure 11, Π″ can be treated as a simplified version of Π* in Figure 3. In Π″, although the device *A* and the device *B* reuse the shared *DHKey* in their pairing information tables, they still need to randomly generate *PKa* and *PKb* and then exchange them during the public key exchange phase. Note that *PKa* and *PKb* are directly selected from the defined elliptic curve [3,5] but are not computed from *SKa* and *SKb* as in Π* and Π′. The authentication stage in Π″ is similar to the authentication stage 2 in Π*. The only difference from the authentication stage is that the device *A* and the device *B* generate *Na* and *Nb* respectively and then exchange them (step 2a, step 2b, step 3, and step 4), that is, these steps appeared in the authentication stage 1 in Π*.

## 5. Privacy Analysis

### 5.1. Threats

Π′s messages are transmitted over the wireless LE channel and therefore are easily tapped by the adversary. Moreover, the possibility of the message loss over the LE channel is higher than that over the wired channel due to mobile system malfunction or wireless communication errors. Hence, we assume that the devices and their LE channel can be controlled by the adversary. In detail, we classify two types of adversaries as follows:

- *Passive adversary (PA)*. A PA eavesdrops on all messages generated by the run of Π. The PA tries to find out *LTK* or other useful information of the targeted devices. However, the PA cannot tamper with any message during the run of Π.
- *Active adversary (AA)*. Besides acting as a PA, an AA can additionally inject, modify, and block the messages transmitted by the devices. When the devices run Π, the AA may impersonate a device by sending his fabricated messages.

According to the security and privacy requirements [1,2,3,5,6], we summarize that Π should be particularly able to defeat against the following attacks:

1. Eavesdropping attack. During the run of Π, the PA or AA eavesdrops on the devices’ messages over the LE channel and tries to derive LTK. When LTK is revealed, the PA can decrypt the encrypted data from the devices and the AA may perform other enhanced attacks, such as the replay attack and the spoofing attack.
2. MITM attack. The MITM attack occurs, when a user wants to connect two devices but instead of connecting directly with each other they unknowingly connect to the AA’s device that plays the role of the device they are attempting to pair with. To achieve the MITM attack, the AA may replay the message(s) in the previous run of Π or forge the message(s) for the current run of Π.
3. Message loss. When the AA blocks the LE service by jamming, the messages transmitted by the devices can be interrupted. During the run of Π, the message loss may cause the data de-synchronization in the pairing information tables. We call it as the de-synchronization attack.
4. Tracking. The PA or AA may track the devices by observing their protocol interactions. For perfect untraceability, Π must ensure that the messages emitted by the targeted device should not be distinguishable from the messages emitted by other devices.

The LESSP protocol makes use of the association models and the ECDH algorithm to prevent the eavesdropping attack and the MITM attack. In Part I [31], we show that the SSP protocols can withstand the eavesdropping attack and the MITM attack under the formal security model. In fact, with the similar security model, (Π′, Π″) described in Section 4 can be proved secure against the eavesdropping attack and the MITM attack. For simplicity, we do not discuss this security feature of (Π′, Π″), because it can be similarly analyzed but nevertheless with a lot of background knowledge of our security model [31]. Next, we propose a formal privacy model to address the de-synchronization attack and the tracking attack.

### 5.2. Privacy Model

Let *k* or 1*^k^* be the security parameter. A real-valued function *f*: *N* → [0, 1] is negligible if for every polynomial *pl*() there exists an integer *N* such that for every *k* > *N*, *f*(*k*) < 1/*pl*(*k*) holds. In the following, we denote an arbitrary negligible function by *negl*. For the sake of readability, we concentrate on asymptotic complexities and privacy, although all our results can be written with more precise bounds.

The Bluetooth pairing system includes a polynomial number of the devices named as 1, 2, …, and *n*. Each device is a probabilistic interactive Turing machine with an independent source of randomness and unlimited internal storage. Any different devices *A*, *B* ∈ {1, 2, …, *n*} are allowed to run a Π multiple times with the polynomial bound. The goal of the PA and AA is to identify the targeted device by exploiting the runs of Π among the devices. Our formal privacy model generally simulates the pairing process of the devices controlled by the adversary and excludes the potential attacks using the privacy definition. Our model can be slightly adjusted to fit any particular deployment model of the Bluetooth pairing system.

#### 5.2.1. Modeling Adversarial Power

To compromise the privacy of Π, the adversary is able to access the Π′s instances run by the devices. This adversarial behavior is formalized by allowing the adversary to interact freely with a group of oracles. The oracles provided for the adversary are defined as follows:

1. Init (*A*, *B*): This oracle call initializes the fresh partnered instances Π*^A^*^,*B*^ and Π*^B^*^,*A*^, where 1 ≤ *A* ≤ *n*, 1 ≤ *B* ≤ *n*, and *A* ≠ *B*. For convenience, we assume that *A* is always the initiating device and *B* is always the non-initiating device. For (Π′, Π″), the Init oracle obeys the following rule. If either the device *A* or the device *B* cannot find the counterpart’s device address in its pairing information table, two fresh instances Π′*^A^*^,*B*^ and Π′*^B^*^,*A*^ are initialized; else two fresh instances Π″*^A^*^,*B*^ and Π″*^B^*^,*A*^ are initialized.
2. Send (Π*^A^*^,*B*^, *M*) or Send (Π*^B^*^,*A*^, *M*): When this Send oracle is called, it sends the message *M* to *A*’s Π*^A^*^,*B*^ or *B*’s Π*^B^*^,*A*^. The output of the Send oracle is whatever message Π*^A^*^,*B*^ or Π*^B^*^,*A*^ would send after receiving the message *M* according to the current run progress of Π*^A^*^,*B*^ or Π*^B^*^,*A*^. Especially, the Send oracle outputs the first message of the current run of Π, if Send (Π*^A^*^,*B*^, null) is invoked and Π*^A^*^,*B*^ is a new instance. The Send oracles simulate the AA by injecting the messages into the runs of Π or modifying the messages transmitted by the devices during the runs of Π. Moreover, the device should terminate the current run of Π, if the message *M* into the Send oracle is invalid. Hence, the Send oracle can also simulate the AA by blocking the runs of Π by sending the error messages.
3. Execute (*A*, *B*): When this Execute oracle is called, a complete run between the partnered instances Π*^A^*^,*B*^ and Π*^B^*^,*A*^ is performed. The output of the Execute oracle is a transcript of Π, i.e., the complete series of messages exchanged by the partnered instances Π*^A^*^,*B*^ and Π*^B^*^,*A*^. The Execute oracle simulates the PA’s ability to passively eavesdrop on the protocol runs. To protect the user privacy, the adversary should learn nothing of the device from such oracle calls.
4. Result Π*^A^*^,*B*^ or Result (Π*^B^*^,*A*^): If Π*^A^*^,*B*^ or Π*^B^*^,*A*^ completes successfully, the output of the Result oracle returns 1; else the output of the Result oracle results 0. The adversary may have access to the partial protocol output, e.g., the observation whether a Bluetooth-activated door opens or not. The Result oracle therefore simulates the adversary knowing whether the device succeeds to be paired with the counterpart device.
5. Reveal Π*^A^*^,*B*^ or Reveal (Π*^B^*^,*A*^): This call outputs the link keys, i.e., *MacKey* and *LTK*, in the device *A*’s Π*^A^*^,*B*^ or the device *B*’s Π*^B^*^,*A*^. If *MacKey* and *LTK* do not exist, the Reveal oracle outputs a null. This Reveal oracle simulates the improper exposure of the link keys. To defeat tracking, the protocol needs to ensure independence of the link keys in different instances.
6. Corrupt (Π*^A^*^,*B*^) or Corrupt (Π*^B^*^,*A*^): This call outputs *DHKey* in Π*^A^*^,*B*^ or Π*^B^*^,*A*^, if it exists. Otherwise, the Corrupt oracle outputs a null. This Corrupt oracle simulates the improper exposure of the Diffie-Hellman key.
7. Test (*A*, *B*): This call is used to define the privacy of Π and does not simulate any real adversarial ability. The adversary is only allowed to query the Test oracle once. To answer the query, the Test oracle flips a fair random coin *b* ∈ {0, 1}. If *b* = 0, the Test oracle calls Execute (*A*, *B*) and returns the output of Execute (*A*, *B*) to the adversary; else the Test oracle itself generates a random transcript of Π and returns it to the adversary. Here, we demand that the random transcript of Π has the same bit length with that of the output of Execute (*A*, *B*). The adversary is required to guess the random bit *b* according to the output of Test (*A*, *B*). For (Π′, Π″), the Test oracle is specifically implemented as follows.

When *b* = 0, the Test oracle calls Execute (*A*, *B*). To be specific, the device *A* and the device *B* respectively search their own pairing information tables. If the other device addresses exist in their tables, then Π″ is run and the complete series of the corresponding transmitted messages is output. Otherwise, the complete series of the corresponding transmitted messages is output through the run of Π′.

When *b* = 1, the Test oracle generates *SKa*, *PKa*, *SKb*, *PKb*, *Na*, *Nb*, *Ea*, and *Eb* according to the specification of Π′ and returns *PKa*, *PKb*, *Na*, *Nb*, *Ea*, and *Eb* to the adversary.

#### 5.2.2. Defining Privacy

In our privacy model, violating the privacy of Π informally means that the adversary distinguishes a transcript of Π generated by a device from a random transcript without any relation to the device. It is reminiscent of the polynomially indistinguishable chosen-plaintext attack [32] in a cryptosystem security game.

Let 𝒜 denote the adversary. As shown in Table 2, we present a privacy game 𝒫𝒢_𝒜,Π_[*n*, *k*], where *n* is the number of the devices in the Bluetooth pairing system and *k* is the security parameter of Π. The Bluetooth pairing system is set up in the system setup phase. Each device in the Bluetooth pairing system uses Π to pair with each other. During the learning phase, 𝒜 is allowed to invoke the Init, Send, Execute, Result, Reveal, and Corrupt oracles defined in Section 5.2.1. Here, assume that 𝒜 has the polynomial limits of its computational power and the polynomial bounds of the number of oracles calls. In the challenge phase, 𝒜 arbitrarily chooses two devices, i.e., *A* and *B*, without the compromise of their shared *DHKey*, and then calls Test (*A*, *B*). The task of 𝒜 is to guess the random bit *b* according to the output of Test (*A*, *B*). 𝒜 should determine *b* by distinguishing the transcript of Π, i.e., the output of Execute (*A*, *B*), from a random transcript generated by the Test oracle. Let 𝒜[*n*, *k*] denote 𝒜 with *n* and *k*. Π is defined to be private if no 𝒜[*n*, *k*] has a non-negligible advantage in successfully guessing the random bit *b* in the Test oracle. Now, the privacy of Π can be defined as follows.

**Definition 1** **(k-Privacy).** *Π is k-privacy if for any probabilistic polynomial time 𝒜[n, k] there exists a negligible function negl such that:*

(1) $$Prob({𝒫𝒢}_{𝒜,\Pi }[n,k]succeeds\;in\;guessing\;b)<\frac{1}{2}+negl(k).$$

We show that the LESSP protocol fails to pass the privacy evaluation under the above privacy game. Consider 𝒫𝒢_𝒜,Π*_[*n*, *k*]. During the learning phase, the adversary 𝒜 is able to select any two different devices *A*, *B* ∈ {1, 2, …, *n*}, call the oracle Execute (*A*, *B*), and obtain its output, i.e., *PKa*, *PKb*, *Na*, *Nb*, *Ea*, and *Eb*. During the challenge phase, 𝒜 continues to select two uncorrupted devices *A* and *B*, and then calls the oracle Test (*A*, *B*). The oracle Test (*A*, *B*) returns *PKa*, *PKb*, *Na*, *Nb*, *Ea*, and *Eb* to 𝒜. If the previous *PKa* in the learning phase is equal to the current *PKa*, 𝒜 determines *b*′ = 0 in step 6 of 𝒫𝒢_𝒜,Π*_[*n*, *k*]; else 𝒜 guesses *b*′ = 1 in step 6 of 𝒫𝒢_𝒜,Π*_[*n*, *k*]. Assume that p^*^ is the probability of reusing (*SKa*, *PKa*) in the device *A*’s next run of Π*. We have:

(2) $$\begin{array}{c} Prob({𝒫𝒢}_{𝒜,\Pi^{*}}[n,k]\;\mathrm{succeeds}\;\mathrm{in}\;\mathrm{guessing}\;b)=\\ Prob({𝒫𝒢}_{𝒜,\Pi^{*}}[n,k]\;\mathrm{succeeds}\;\mathrm{in}\;\mathrm{guessing}\;b\;|\;b=0)\;Prob(b=0)+\\ Prob({𝒫𝒢}_{𝒜,\Pi^{*}}[n,k]\;\mathrm{succeeds}\;\mathrm{in}\;\mathrm{guessing}\;b\;|\;b=1)\;Prob(b=1)\approx p^{*}\frac{1}{2}+\frac{1}{2}=\frac{1}{2}+\frac{p^{*}}{2}. \end{array}$$

Because p^*^ is not negligible, we conclude that Π* violates the privacy requirement in Definition 1.

### 5.3. Privacy Result of Our Improvement and Its Proof

The adversary may initiate the de-synchronization attack on the device and further observe the abnormal run of Π to track this device. In (Π′, Π″), the only way for the attack to be successful is to de-synchronize the data of the pairing information tables between the pairing devices. However, in Figure 9, we see that the devices should implement Π′, if a device fails to share *DHKey* in the previous run. Clearly, (Π′, Π″) can prevent the adversary from guessing the random bit *b* by the de-synchronization attack, because it does not make the device enter the abnormal run. Hence, we do not consider the de-synchronization attack in the following discussion. We need two well-known cryptographic assumptions to prove the privacy result of (Π′, Π″). For the reader’s convenience, we rewrite them as follows:

**Definition 2** **(Definition 3 in [31]).** *Let gen(1^k^) be a parameter generation algorithm that outputs the description of a group G, its generator P ∈ G, and its order q. We say the Decisional Diffie-Hellman (DDH) problem is hard relative to G if for all probabilistic polynomial time algorithms D there exists a negligible function negl such that:*

|*Prob*(*D* (*gen* (1^*k*^), *a*·*P*, *b*·*P*, *ab*·*P*) = 1) − *Prob*(*D* (*gen*(1^*k*^), *a*·*P*, *b*·*P*, *c*·*P*) = 1)| < *negl*(*k*), (3)

*where a, b, and c are randomly chosen in {1, …, q}.*

**Definition** **3.** Let gen(1^k^) be a parameter generation algorithm that outputs the description of a group G, its generator P ∈ G, and its order q. Let H_0_() be a key derivation function. Let H_1_(H_0_(ab·P, ), ) be a keyed pseudorandom function using the Diffie-Hellman key derivation function if for every probabilistic polynomial time distinguisher D* there exists a negligible function negl such that

|*Prob*(D*(*gen*(1^*k*^), *a*·*P*, *b*·*P*, *H*_1_(*H*_0_(*ab*·*P*, ), )) = 1) − *Prob*(D*(*gen*(1^*k*^), *a*·*P*, *b*·*P*, *R*()) = 1)| < *negl*(*k*), (4)

*where a and b are randomly chosen in {1, …, q} and R() is a truly random function.*

Definition 3 is almost the same as Definition 4 in [31]. However, we replace the Diffie-Hellman key *ab·P* with a key derivation function *H*_0_(*ab·P*, ). We have *MacKey* || *LTK* = *f*5(*DHKey*, *Na*, *Nb*, *A*, *B*) in step 5a and step 5b of Π*, Π′, and Π″. Hence, we define *MacKey* = [*f*5(*DHKey*, *Na*, *Nb*, *A*, *B*)]_Mac_. To reduce our privacy results, the following lemma is required.

**Lemma** **1.** *Prob( 𝒫𝒢_𝒜,Π′_[n, k] succeeds in guessing b) = *½*.*

**Proof of** **Lemma 1.** As shown in Figure 10, we know (*SKa*, *PKa*) and (*SKb*, *PKb*) are updated in each run of Π′ and any (*SKx*, *PKx*) in Π′ is independent from each other. Hence, during the learning phase of the privacy game 𝒫𝒢_𝒜,Π′_, 𝒜 learns no knowledge of the bit *b* by calling the Reveal and Corrupt oracles. It means that we can exclude the Reveal and Corrupt oracles in 𝒫𝒢_𝒜,Π′_.

In the learning phase of 𝒫𝒢_𝒜,Π′_, 𝒜 can call the Init, Send, Execute, and Result oracles to any different pairing devices *i*, *j* ∈ {1, 2, …, *n*} and then receive the corresponding responses. Next, 𝒜 enters into the challenge phase of 𝒫𝒢_𝒜,Π′_. He selects a challenge device *A* ∈ {1, 2, …, *n*} and any other device *B* ∈ {1, 2, …, *n*} and calls the oracle Test (*A*, *B*). We know that Test oracle acts as follows.

When *b* = 0, it calls Execute (*A*, *B*) and returns its output *PKa*, *PKb*, *Na*, *Nb*, *Ea*, and *Eb* to 𝒜. Here, according to the specification of Π′, *PKa*, *PKb*, *Na*, *Nb*, *Ea*, and *Eb* are respectively generated by the device *A* and the device *B*.

When *b* = 1, the Test oracle directly generates its *PKa*, *PKb*, *Na*, *Nb*, *Ea*, and *Eb* according to the specification of Π′ and returns them to 𝒜.

Whether the random bit *b* is 0 or 1, 𝒜 always receives a uniform transcript of Π′, i.e., *PKa*, *PKb*, *Na*, *Nb*, *Ea*, and *Eb*. Hence, we have:

(5) $$Prob({𝒫𝒢}_{𝒜,\Pi {}^{\prime }}[n,\;k]\;\mathrm{succeeds}\;\mathrm{in}\;\mathrm{guessing}\;b)\;=\;\frac{1}{2}.$$

This completes the proof. □

**Theorem** **1.** *Assume that P256() in both Π′ and Π″ satisfies the DDH assumption as in Definition 2. Assume that [f5()]_Mac_ and f6() in Π′ and Π″ are respectively the key derivation function and the keyed pseudorandom function using the Diffie-Hellman key derivation function as in Definition 3. Assume that the device always keeps its pairing information table secret. Then, (Π′, Π″) is private according to Definition 1.*

**Proof of** **Theorem 1.** We know that the Corrupt oracle cannot help 𝒜 to guess the random bit *b* in the Test oracle of the privacy game 𝒫𝒢_𝒜,(Π′,Π″)_, because all *DHKeys* generated by Π′ are random and independent, the pairing information table is kept secret, and the oracles Corrupt (Π*^A^*^,*B*^) and Corrupt (Π*^B^*^,*A*^) are not allowed if the corresponding *DHKey* is used during the challenge phase of 𝒫𝒢_𝒜,(Π′,Π″)_. Therefore, we exclude the Corrupt oracle in the following discussions.

To violate the privacy of (Π′, Π″), 𝒜 completely relies on the correct guess of the random bit *b* after the Test oracle is called during the challenge phase. When the Test oracle calls Execute (*A*, *B*), two devices *A* and *B* run either Π′ or Π″ if *b* = 0. There are therefore two cases to consider:

Case 1: Π′ is run in the Test oracle when *b* = 0. This case means that both the device *A* and the device *B* do not maintain the shared *DHKey* in their pairing information tables. When *b* = 0, the Test oracle calls Execute (*A*, *B*) and returns the output of Execute (*A*, *B*) to 𝒜. Here, Execute (*A*, *B*) outputs a newly exchanged transcript of Π′. When *b* = 1, the Test oracle also simulates Π′ and returns its random transcript of Π′ to 𝒜. Let *E*_1_ be the event that 𝒫𝒢_𝒜,(Π′,Π″)_ [*n*, *k*] succeeds in guessing *b*, if Π′ is run in the Test oracle when *b* = 0. By Lemma 1, we have *Prob*(*E*_1_) = *Prob*( 𝒫𝒢_𝒜,Π′_[*n*, *k*] succeeds in guessing *b*) = ½.

Case 2: Π″ is run in the Test oracle when *b* = 0. This case means that both the device *A* and the device *B* maintain the shared *DHKey* in their pairing information tables. During the learning phase of 𝒫𝒢_𝒜,(Π′,Π″)_, 𝒜 can collect and verify the transcripts of (Π′, Π″), send the fabricated messages, and disclose the secret keys by calling the Execute, Result, Init, Send, and Reveal oracles. Certainly, 𝒜 can implement these operations for the device *A* and the device *B*. And then, during the challenge phase of 𝒫𝒢_𝒜,(Π′,Π″)_, 𝒜 must strictly depend on correctly guessing the random bit *b* in the oracle Test (*A*, *B*). Let *E*_2_ be the event that 𝒫𝒢_𝒜,(Π′,Π″)_ [*n*, *k*] succeeds in guessing *b*, if Π″ is run in the Test oracle when *b* = 0. If (Π′, Π″) is private under 𝒫𝒢_𝒜,(Π′,Π″)_ [*n*, *k*], we know that:

(6) $$Prob(E_{2})<\frac{1}{2}+negl(k).$$

Let *δ*_1_(*k*) be a function such that

(7) $$Prob(E_{2})<\frac{1}{2}+\delta_{1}(k).$$

We demonstrate that *δ*_1_(*k*) is negligible by presenting a DDH problem distinguisher *D*_1_ with the same advantage *δ*_1_(*k*). The distinguisher *D*_1_ receives (*a·P*, *b·P*, *K*) and attempts to determine whether *K* = *ab·P* or *K* is a random element in the group *G*. The distinguisher *D*_1_ simulates the learning phase and the challenge phase of 𝒫𝒢_𝒜,(Π′,Π″)_ in the following:

Simulation of the learning phase. When 𝒜 calls the Execute, Result, Init, Send, and Reveal oracles, the distinguisher *D*_1_ also invokes these oracles to the corresponding devices and returns the outputs of these oracles to 𝒜.

Simulation of the challenge phase. When 𝒜 selects the device *A* and the device *B* and calls the oracle Test (*A*, *B*), the distinguisher *D*_1_ simulates the Test oracle as follows:

The distinguisher *D*_1_ flips a fair random coin *b*.

If *b* = 0, the distinguisher *D*_1_ calls Execute (*A*, *B*). It means that a complete run between the partnered instances Π″*^A^*^,*B*^ and Π″*^B^*^,*A*^ is performed. The output of the Execute oracle is the complete series of messages exchanged by the partnered instances Π″*^A^*^,*B*^ and Π″*^B^*^,*A*^, i.e., *PKa*, *PKb*, *Na*, *Nb*, *Ea*, and *Eb*. Note that both the device *A* and the device *B* obtain the shared *DHKey* in their pairing information tables and the shared *DHKey* has no relation with *PKa* and *PKb*. The distinguisher *D*_1_ returns *PKa*, *PKb*, *Na*, *Nb*, *Ea*, and *Eb* to 𝒜.

If *b* = 1, the distinguisher *D*_1_ sets *PKa* = *a·P* and *PKb* = *b·P*, randomly generates *Na* and *Nb*, and computes *MacKey* || *LTK* = *f*5(*K*, *Na*, *Nb*, *A*, *B*), *Ea* = *f*6(*MacKey*, *Na*, *Nb*, *rb*, *IOCapA*, *A*, *B*), and *Eb* = *f*6(*MacKey*, *Nb*, *Na*, *ra*, *IOCapB*, *B*, *A*). The distinguisher *D*_1_ returns its *PKa*, *PKb*, *Na*, *Nb*, *Ea*, and *Eb* to 𝒜.

After the query of the Test oracle, the distinguisher *D*_1_ outputs 1 when 𝒜 outputs his guessing *b*′ = *b* in 𝒫𝒢_𝒜,(Π′,Π″)_. In the view of 𝒜, the above simulation by the distinguisher *D*_1_ is exactly 𝒫𝒢_𝒜,(Π′,Π″)_ when *K* = *ab·P.* Hence, we know:

(8) $$Prob(D_{1}(\mathrm{gen}(1^{k}),\;a\cdot P,\;b\cdot P,\;\mathrm{ab}\cdot P)\;=\;1)<\frac{1}{2}+\delta_{1}(k).$$

When *K* is a random element in the group *G*, *MacKey* is computed by using the random element *K* instead of *DHKey* = *ab·P* and *Ea* and *Eb* are further calculated by using *MacKey*. We know that 𝒜 cannot guess the random bit *b* from *PKa* and *PKb*, because the device *A*, the device *B*, and the distinguisher *D*_1_ all does not use *PKa* and *PKb* to generate *DHKey* in this situation. Let *δ*_2_(*k*) be a function such that 𝒜 outputs *b*′ = *b* with probability ½ + *δ*_2_(*k*), where *δ*_2_(*k*) is the advantage by exploiting *MacKey*, *Ea*, and *Eb*. We show that *δ*_2_(*k*) is a negligible function by contradiction. Assume that *δ*_2_(*k*) is a non-negligible function. We assume that *Ea* and *Eb* are generated by the truly random function *R*() if the random element *K* is replaced with *DHKey*. We directly construct the distinguisher *D*_2_ to tell the keyed pseudorandom function *f*6([*f*5(*DHKey*, )]_Mac_, ) from the truly random function *R*(). And the distinguisher *D*_2_ outputs a correct guess with probability ½ + *δ*_2_(*k*) by the same reason as the distinguisher *D*_1_ does in the case where the random element *K* exists. However, the advantage of the distinguisher *D*_2_ is a non-negligible function *δ*_2_(*k*), which is contradiction against Definition 3. Hence, *δ*_2_(*k*) is a negligible function. We further have:

(9) $$\begin{array}{c} \left|Prob(D_{1}(gen(1^{k}),a\cdot P,b\cdot P,ab\;P)=1)-Prob(D_{1}(gen(1^{k}),a\cdot P,b\cdot P,K)=1)\right|\\ =\;|\frac{1}{2}+\delta_{1}(k)\;-\;\frac{1}{2}\;-\;\delta_{2}(k)|\;=\;|\;\delta_{1}(k)\;-\;\delta_{2}(k)|. \end{array}$$

By Definition 2, we know that *δ*_1_(*k*) is a negligible function due to the negligible function *δ*_2_(*k*). Assume that *p*′ denotes the probability of *E*_1_ occurring and *p*″ denotes the probability of *E*_2_ occurring. Clearly, it follows that *p*′ + *p*″ = 1. We further have:

(10) $$\begin{array}{c} Prob({𝒫𝒢}_{𝒜,(\Pi^{\prime },\Pi^{\prime \prime })}\;\mathrm{succeeds}\;\mathrm{in}\;\mathrm{guessing}\;b)=Prob(E_{1})p^{\prime }+Prob(E_{2})p^{\prime \prime }<\\ \frac{1}{2}p{}^{\prime }+(\frac{1}{2}+\delta_{1}(k))p{}^{\prime \prime }\;=\;\frac{1}{2}+\delta_{1}(k)p{}^{\prime \prime }. \end{array}$$

Thus, we conclude that (Π′, Π″) is private by Definition 1. □

## 6. Performance Evaluation

In this section, we discuss the implementation efficiency of our improvement due to its LE applications. Our performance evaluation has two levels, i.e., the basic protocol analysis and the Bluetooth pairing system analysis. We know that it is not easy to measure the costs of the human operations in the association models. Hence, we omit the costs of these models in all pairing protocols. As shown in Figure 3, Figure 10 and Figure 11, the costs of both pairing devices are all the same in these protocols, if we do not consider the association models. Therefore, we always calculate the costs on one device. The experiments are executed on the following platform:

(1) Experiment environment: The basic cryptographic algorithms are executed on Windows 10 64 bits, Intel(R) Core(TM) i7-8700 CPU @3.20 GHz 3.19GHz, and 8.00 GB RAM.
(2) Cryptographic tool: Python 3.6.6 cryptography toolkit PyCryptodemo.

### 6.1. Basic Protocol Analysis

#### 6.1.1. Computation Cost

**Figure 12:**
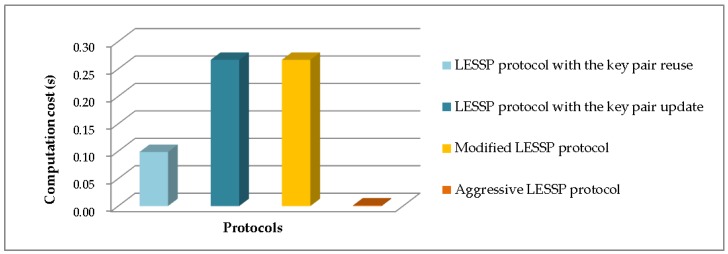
Experiment comparison of computation time costs.

Table 3 shows the computation costs of the LESSP protocol and our improved one. The LESSP protocol is further divided into two cases, i.e., the key pair reuse and the key pair update. The experiment results of these pairing protocols are shown in Figure 12. We can see that the computation efficiency of the aggressive LESSP protocol is significantly higher than other protocols.

#### 6.1.2. Communication Cost and Storage Cost

As shown in Table 4, we calculate the communication costs of the transmitted messages during the runs of the pairing protocols. And for the storage cost, we merely calculate the long-term parameters, i.e., the reused key pair and the values stored in the pairing information table.

#### 6.1.3. Energy Cost Concern

The energy cost of our improvement is the same as or less than the LESSP protocol, if the cost of searching the pairing information table is free. The reason is that our aggressive LESSP protocol does not need to generate the ECDH private-public key pair and compute the Diffie-Hellman key and other computation and communication costs of both our improvement and the LESSP protocol are the same.

### 6.2. Bluetooth Pairing System Analysis

We consider the computation costs of a device, which probably pairs with a set of other n* devices in the Bluetooth pairing system. Let *P* be the total number of the pairing run and *m* be the number of the records of the Bluetooth device’s pairing information tables in our improvement. Let *W* be the probability distribution of the devices’ pairing runs, where *W* = [*p*_1_, *p*_2_, …, p_n__*_] and *p*_1≤_*_i_*_≤n*_ denotes the probability of the event that the device pairs with the device *i* in the Bluetooth pairing system. We know that *p*_1_ + *p*_2_ + … + p_n__*_ = 1.

#### 6.2.1. Number of Devices

**Figure 13:**
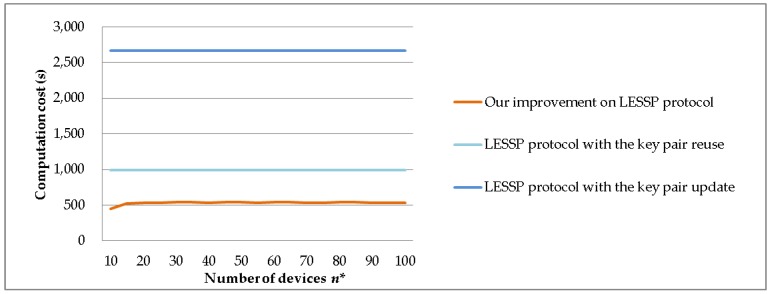
The computation time costs against the number of devices n*.

We set *P* = 10,000 and *m* = 5 and assume *p*_1_ + *p*_2_ + *p*_3_ + *p*_4_ + *p*_5_ = 0.8 and *p*_6_ + *p*_7_ + … + p_n__*_ = 0.2. We evaluate the computation costs of the LESSP protocol and our improvement, when the number of devices n* = 10, 15, 20, 25, 30, 35, 40, 45, 50, 55, 60, 65, 70, 75, 80, 85, 90, 95, and 100, respectively. Figure 13 shows the experimental results of the device’s computation time costs. Our improvement yields about 100–400% time reduction against the LESSP protocol with the key pair reuse and the LESSP protocol with the key pair update.

#### 6.2.2. Number of Records

**Figure 14:**
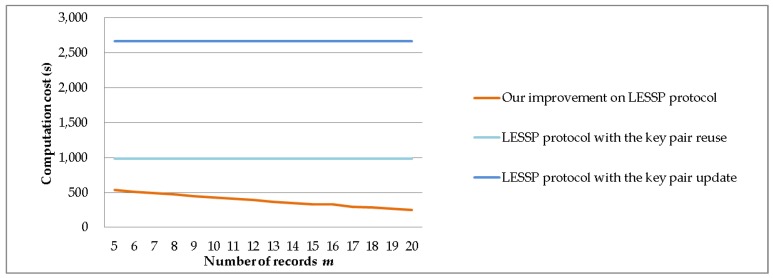
The computation time costs against the number of records *m*.

We set n* = 50 and *P* = 10,000 and assume *p*_1_ + *p*_2_ + *p*_3_ + *p*_4_ + *p*_5_ = 0.8 and *p*_6_ + *p*_7_ + … + *p*_50_ = 0.2. We evaluate the computation costs of the LESSP protocol and our improvement, when the number of records *m* ranges from 5 to 20. Figure 14 shows the experimental results of the device’s computation time costs. In Figure 14, we can observe that the time saving of our improvement is similar to the case of the variable number of devices in Section 6.2.1.

#### 6.2.3. Probability Distribution

**Figure 15:**
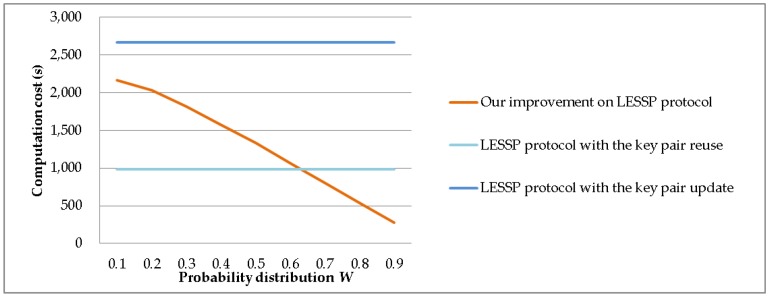
The computation time costs against probability distribution *W*.

We set n* = 50, *P* = 10,000, and *m* = 5. We evaluate the computation costs of the LESSP protocol and our improvement, when the probability distribution *W* = [*p*_1_, *p*_2_, …, *p*_50_] varies in our improvement. The value range of *p*_1_ + *p*_2_ + *p*_3_ + *p*_4_ + *p*_5_ changes from 0.1 to 0.9. Figure 15 shows the device’s experimental time costs. When the value of *p*_1_ + *p*_2_ + *p*_3_ + *p*_4_ + *p*_5_ increases, the time cost of our improvement significantly reduces. In fact, in wireless personal area networks, it is common for a few devices to have very high pairing probabilities.

We believe that the execution performance of both our improvement and the LESSP protocol should be similar as that observed in Figure 13, Figure 14 and Figure 15, when they are realized on commercial-off-the-shelf (COTS) LE Bluetooth devices. That is to say, although COTS Bluetooth LE devices may have different processing power, the execution performance is dominated by the corresponding parameters in Section 6.2.1, Section 6.2.2 and Section 6.2.3, whereas COTS Bluetooth LE devices merely determine the detailed values of the computation costs.

## 7. Conclusions and Future Work

When the LESSP protocol is deployed in the Bluetooth system, an adversary can track the targeted device by its reuse of the private-public key pair. In fact, the SSP protocol has a similar privacy weakness. Our improvement on the LESSP protocol is proven to be able to overcome the privacy weakness under the proposed privacy model. Moreover, the experimental results show that our improvement is as efficient as the LESSP protocol.

It needs to be pointed out that our improvement has not been evaluated with open-source software stacks and software-defined radios. Therefore, our future work is to use the Universal Software Radio Peripheral (USRP) [33] to implement the LESSP protocol and our improvement. Based on it, we can obtain the energy cost and the more accurate efficiency results for these protocols. We conclude with two interesting open problems for the privacy of the LESSP protocol. Firstly, our work has not dealt with the privacy of the association models in the LESSP protocol. In fact, the adversary may violate the privacy of the LESSP protocol by intercepting the communications during the run of the association models. Secondly, we have not investigated the forward/backward privacy of the LESSP protocol. Backward privacy means that, if the adversary reveals the secret of a Bluetooth device *A* at time *τ*, the adversary is not able to tell whether a run of the protocol before time *τ* involves the device *A*. Forward privacy deals with the adversary who, even with the knowledge of the secret of the device *A* at time *τ*, cannot determine whether the same device was involved in a run of the protocol that occurred at time *τ* + *τ*′ (for some *τ*′ > 0). To solve these two privacy problems, we need a new privacy model and a redesigned LESSP protocol.

## Figures and Tables

**Table 1:** Description of notation.

Term	Definition
Π*	LESSP protocol
Π′	Modified LESSP protocol
Π″	Aggressive LESSP protocol
(Π′, Π″)	Our improvement on Π*
Π	One of Π*, (Π′, Π″), and other LESSP-type protocol
Π*^A^*^,*B*^	Bluetooth device *A*’s any Π instance run with Bluetooth device *B*
*X*	Bluetooth address of Bluetooth device *X*
*IOcapX*	IO capabilities of Bluetooth device *X*
(*SKx*, *PKx*)	ECDH private-public key pair of Bluetooth device *X*
*DHKey*	Diffie-Hellman key
*MacKey*	128-bit key of message authentication code
*LTK*	128-bit long term key used to generate the contributory session key for an encrypted connection
*Nx*	Nonce (unique random value) generated by Bluetooth device *X*
*Ex*	Check value from Bluetooth device *X*
*rx*	Value from Bluetooth device *X*
*P*256()	ECDH algorithm used to compute *DHKey*
*f*5()	Function used to compute *MacKey* and *LTK*
*f*6()	Function used to generate *Ex*
*Prob*(*E*)	Probability of event *E* occurring
*Prob*(*E*′ | *E*″)	Conditional probability of event *E*′ given event *E*″, which measures probability of event *E*′ occurring, given that event *E*″ has occurred

**Table 2:** Bluetooth privacy game for the LESSP-type protocols.

Untraceability on Bluetooth Device
**Bluetooth Privacy Game 𝒫𝒢_𝒜,Π_[*n*, *k*]**
**Phase 1. System setup**
Select a polynomial number of the Bluetooth devices named as 1, 2, …, and *n*. Determine the bit length of *DHKey* denoted as *k*_1_ and the bit length of *MacKey* denoted as *k*_2_. Let *k* = min( *k*_1_, *k*_2_) be the security parameter. (Optional) Set the available storage capacity for the pairing information table in the Bluetooth device.
**Phase 2. Learning**
3. For any Bluetooth device in {1, 2, …, *n*}, the adversary 𝒜 can call the oracles defined in Section 5.2.1. The following oracles are polynomially invoked with interleaved order. 3.1. 𝒜 calls the oracles *O*_PA_ = {Execute, Result} to act as the PA. 3.2. 𝒜 calls the oracles *O*_AA_ = {Init, Send, Reveal, Corrupt} to act as the AA.
**Phase 3. Challenge**
4. 𝒜 selects a challenge Bluetooth device *A* ∈ {1, 2, …, *n*} and any other Bluetooth device *B* ∈ {1, 2, …, *n*}. Here, the oracle Corrupt (Π*^A^*^,*B*^) or the oracle Corrupt (Π*^B^*^,*A*^) is not allowed during the learning phase, if the compromised *DHKey* exists in both devices’ pairing information tables. 5. 𝒜 calls the oracle Test (*A*, *B*). 6. 𝒜 outputs his guessing bit *b*′.
𝒜 wins if *b* = *b*′.

**Table 3:** Computation efficiency of pairing protocols.

Protocol	Computation Cost
LESSP protocol with the key pair reuse	*P*256 ^1^ + *Rand* ^2^ + 3*AES*-*CMAC*_128_^3^
LESSP protocol with the key pair update	*GenKP*^4^ + *P*256 + *Rand* + 3*AES*-*CMAC*_128_
Modified LESSP protocol	*GenKP* + *P*256 + *Rand* + 3*AES*-*CMAC*_128_
Aggressive LESSP protocol	*GenPK*^5^ + *Rand* + 3*AES*-*CMAC*_128_

^1^*P*256 denotes computation cost of ECDH algorithm; ^2^*Rand* denotes computation cost of 128-bit pseudo-random number generator; ^3^ *AES*-*CMAC*_128_ denotes computation cost of one way cryptographic function; ^4^ *GenKP* denotes computation cost of ECDH key generator; ^5^ *GenPK* denotes computation cost of random elliptic curve point generator in Π″.

**Table 4:** Communication and storage costs in one run of each protocol.

Protocol	Communication Cost (bits)	Storage Cost (bits)
LESSP protocol	1024	512
Modified LESSP protocol	1024	304*m* ^1^
Aggressive LESSP protocol	1024	

^1^*m* denotes the number of the records in the device’s pairing information table in Figure 8.
